# Wise Management of Ovarian Cancer: On the Cutting Edge

**DOI:** 10.3390/jpm10020041

**Published:** 2020-05-21

**Authors:** Stergios Boussios, Christos Mikropoulos, Eleftherios Samartzis, Peeter Karihtala, Michele Moschetta, Matin Sheriff, Afroditi Karathanasi, Agne Sadauskaite, Elie Rassy, Nicholas Pavlidis

**Affiliations:** 1 Medway NHS Foundation Trust, Windmill Road, Gillingham, Kent ME7 5NY, UK; matin.sheriff@nhs.net (M.S.); a.karathanasi@nhs.net (A.K.); agne.sadauskaite@nhs.net (A.S.); 2 AELIA Organization, 9th Km Thessaloniki—Thermi, 57001 Thessaloniki, Greece; 3 St Luke’s Cancer Center, Royal Surrey County Hospital, Egerton Rd, Guildford GU2 7XX, UK; christos.mikropoulos@nhs.net; 4 Division of Gynecology, University Hospital Zurich, Frauenklinikstrasse 10, CH-8091 Zürich, Switzerland; Eleftherios.Samartzis@usz.ch; 5 Department of Oncology, University of Helsinki and Helsinki University Hospital Comprehensive Cancer Center, P.O. Box 100, FI-00029 Helsinki, Finland; Peeter.Karihtala@oulu.fi; 6 Cambridge University Hospitals NHS Foundation Trust, Hills Rd, Cambridge CB2 0QQ, UK; michelemoschetta1@gmail.com; 7 Department of Cancer Medicine, Gustave Roussy Institut, 94805 Villejuif, France; elie.rassy@hotmail.com; 8 Department of Hematology-Oncology, Hotel Dieu de France University Hospital, Faculty of Medicine, Saint Joseph University, Beirut 166830, Lebanon; 9 Medical School, University of Ioannina, Stavros Niarchou Avenue, 45110 Ioannina, Greece; npavlid@uoi.gr

**Keywords:** ovarian cancer, next-generation sequencing, homologous recombination repair, PARP inhibitors, AURK inhibitors, metformin, personalized treatment

## Abstract

Epithelial ovarian cancer (EOC) is the fifth leading cause of cancer mortality among women. Two-thirds of patients present at advanced stage at diagnosis, and the estimated 5 year survival rate is 20–40%. This heterogeneous group of malignancies has distinguishable etiology and molecular biology. Initially, single-gene sequencing was performed to identify germline DNA variations associated with EOC. However, hereditary EOC syndrome can be explained by germline pathogenic variants (gPVs) in several genes. In this regard, next-generation sequencing (NGS) changed clinical diagnostic testing, allowing assessment of multiple genes simultaneously in a faster and cheaper manner than sequential single gene analysis. As we move into the era of personalized medicine, there is evidence that poly (ADP-ribose) polymerase (PARP) inhibitors exploit homologous recombination (HR) deficiency, especially in breast cancer gene 1 and 2 (*BRCA1/2*) mutation carriers. Furthermore, extensive preclinical data supported the development of aurora kinase (AURK) inhibitors in specific tumor types, including EOC. Their efficacy may be optimized in combination with chemotherapeutic or other molecular agents. The efficacy of metformin in ovarian cancer prevention is under investigation. Certain mutations, such as ARID1A mutations, and alterations in the phosphatidylinositol 3-kinase (PI3K)/AKT/mTOR pathway, which are specific in ovarian clear cell carcinoma (OCCC) and endometrioid ovarian carcinoma (EnOC), may offer additional therapeutic targets in these clinical entities. Malignant ovarian germ cell tumors (MOGCTs) are rare and randomized trials are extremely challenging for the improvement of the existing management and development of novel strategies. This review attempts to offer an overview of the main aspects of ovarian cancer, catapulted from the molecular mechanisms to therapeutic considerations.

## 1. Introduction

Approximately 22,440 newly diagnosed cases of ovarian cancer and 14,080 deaths occurred in the United States in 2017 [1]. Only 10% of ovarian cancers are non-epithelial malignant ovarian germ cell tumors (MOGCTs) and sex cord-stromal cell tumors (SCSTs) (5% each). Epithelial ovarian cancer (EOC) differs in epidemiology, etiology, and treatment. Patients diagnosed with EOC should be tested for hereditary susceptibility genes [2]. Beyond germline pathogenic variants (gPVs) in breast cancer genes 1 and 2 (*BRCA1/2*), alterations in *BRIP1*, *RAD51C*, and *RAD51D* and mismatch repair (MMR) genes also enhance EOC risk. Next-generation sequencing (NGS)-based mutation panels profile multiple genes simultaneously, allowing the reporting of numerous genes while saving labor and resources. The identification of homologous recombination (HR)-deficient EOC has significant clinical implications related to the therapeutic choices of either chemotherapeutic or targeted agents. Indeed, patients with *BRCA* mutations or HR deficiency may be treated with maintenance poly (ADP-ribose) polymerase (PARP) inhibitors in first-line settings or at recurrence. Currently, olaparib, rucaparib, and niraparib have been approved by the Food and Drug Administration (FDA) and/or European Medicines Agency (EMA) for the treatment of EOC [3]. Furthermore, aurora kinases (AURKs) are serine/threonine kinases essential for the onset and progression of mitosis and seem to be promising prognostic factors for EOC among other cancers. AURKs have been shown to interact with DNA repair mechanisms and other cell cycle regulators, and could be novel therapeutic targets. Metformin has anti-angiogenic activity in vivo and chemosensitizing effects in vitro in ovarian cancer; nevertheless, epidemiological studies on its use in ovarian cancer are not equally promising as compared with preclinical evidence. The prevalence of ovarian cancer in women with endometriosis is higher than sporadic ovarian cancer. Endometriosis is frequently described in association with ovarian clear cell carcinoma (OCCC) and endometrioid ovarian carcinoma (EnOC). Epigenetics may be implicated in the pathogenesis of endometriosis, whereas DNA methylation serves as an epigenetic biomarker for EOC. Unlike EOC, MOGCTs typically occur in the first three decades of life. Surgery and platinum-based chemotherapy remain the standard of care. Even in advanced-stage disease, patients have a high chance to be cured. MOGCTs rarely develop genetic mutations. The aim of this article is to provide a comprehensive overview of the genetic and therapeutic evolution of EOC and MOGCTs. 

## 2. Genomic Profiling of EOC by NGS

Recently, microarray technologies have been used to elucidate the complexity of genomic alterations of EOC. NGS technology has become widely available to determine a patient’s precise genetic profiling and identify novel mutations for new drug targets. In this context, patients with EOC with *BRCA* mutations or HR deficiency experience therapeutic benefit from platinum agents and PARP inhibitors, whereas immune checkpoint inhibitors are effective in tumors with microsatellite instability [4,5]. Furthermore, the costs to test multiple genes in a pan-cancer panel are lower as compared to sequencing isolated genes sequentially [6]. 

Importantly, multigene panels decrease the chances of missing out on a pathogenic mutation. When a limited number of genes are tested based on clinical indication and results are negative, targetable mutations in untested genes cannot be fully excluded. This risk is even higher in cases of moderate-penetrance genes for which clinical phenotype is less clear, as well as in those without typical presentation or relevant family history [7].

HR is crucial for carcinogenesis. Single-strand DNA breaks (SSBs) are normally repaired by MMR, base excision repair, and nucleotide excision repair, in which PARP1/2 have a key role [8]. Inhibition of these proteins leads to single-strand break accumulation and, consequently to double-strand DNA breaks (DSBs) and cytotoxicity. Unlike PARP2, PARP1 can also mediate the repair of DSBs and regulates the rate of DNA replication fork progression in replication stress [9]. DSBs are repaired either through HR or through non-homologous end joining (NHEJ)—the choice of which is determined by cell cycle phase and chromatin context [10]. NHEJ is the preferred mechanism for repair of DSBs in G1 when a DNA template that could be used for HR is absent. 

HR deficiency can be assessed by the presence of germline and somatic mutations in HR genes using tumor sequencing. However, if mutations in susceptibility genes are ruled out by tumor sequencing, no additional test is required [11]. Furthermore, HR deficiency leads to the occurrence of genomic scars, represented by the loss of heterozygosity (LOH), large-scale state transitions (LSTs), and telomeric allele imbalance (TAI). Two commercial genomic scar assays identify tumors with HR deficiency. “Myriad’s myChoice HRD assay tests for the presence of LOH, TAI, and LSTs across the genome [12]”. A tumor with an HRD score of 42 and above is labeled as HRD positive. The “FoundationFocus™ CDx *BRCA* LOH” detects mutations in the *BRCA1/2* and the percentage of the genome affected by LOH in DNA from tumor tissue samples [13]. Tumors are considered LOH-high if the score is 16 and above. 

In addition to mutations in the *BRCA1/2* genes, genomic alterations involving other genes in HR pathways have been recognized, including Fanconi anemia genes (*BRIP1*, *PALB2*), the core RAD genes (*RAD51C*, *RAD51D*), and genes involved in HR pathways either directly (*CHEK2*, *BARD1*, *NBN*, *ATM*) or indirectly (*cyclin-dependent kinase (CDK) 12*) [4]. However, their real effect over assessment of EOC risk is still uncertain. Genome-wide association studies identified single-nucleotide polymorphisms associated with susceptibility for EOC. The 27 loci associated with invasive EOC identified so far account for 6.4% of the polygenic risk for EOC [14]. 

*RAD51C* loss-of-function gPVs are rare among EOC patients, with their prevalence varying between 0.3% and 1.1% [15]. The lifetime risk of EOC among *RAD51C* carriers is approximately 5% [15]. Both *RAD51C* and *RAD51D* are EOC susceptibility genes that are characterized by a reduced magnitude when compared to *BRCA1/2*. The increased EOC risk for *RAD51C* and *RAD51D* supports their addition to criteria for risk-reducing salpingo-oophorectomy [16]. Furthermore, genetic defects in these genes can function as biomarkers for PARP sensitivity.

Clinical testing for *PALB2* in EOC is not currently recommended. The majority of studies reported relative risks that ranged from 0.9 to 5.5 and lacked statistical power [17]. A study analyzing data from 524 families with *PALB2* gPVs from 21 countries reported that the estimated risk of EOC at age 80 years was 5% (95% CI, 2–10%) [18]. For *PALB2* carriers, risk-reducing surgery should be recommended for cases with strong family history of EOC. The predictive value of *PALB2* is supported by the evidence that *PALB2*-associated tumors respond to platinum-based chemotherapy and PARP inhibitors [4]. 

Several gPVs in the so-called moderate- and low-penetrance genes, such as *BRIP1*, have been reported to be correlated with a moderate lifetime risk of EOC. The prevalence of *BRIP1* gPVs among familial EOC patients can reach 0.7% [19]. The cumulative lifetime risk of EOC diagnosis among *BRIP* mutants has been estimated as 5–5.8% [17], predominantly following menopause. The elevated risk of EOC diagnosis justifies the recommendation for risk-reducing oophorectomies among asymptomatic carriers and this should be guided based on family history and individual’s preference. 

Other hereditary cancer syndromes are also associated with EOC. A lack of *MSH2*, substantial mutations in the *MLH1* or *MSH2* genes, *MLH1*-methylation inactivation, and transcriptional silencing lead to Lynch syndrome [20]. MMR deficiency has been demonstrated in 20–40% of endometrial cancers [21,22], but data on its prognostic value are controversial [21,23,24]. Table 1 reports the frequency and EOC risk of well-established moderate- and high-penetrance susceptibility genes for EOC, whereas Table 2 depicts clinical trials of PARP inhibitors in EOC and their therapeutic potential in patients with HR deficiency. 

Overall, for EOC patients, guidelines recommend testing for moderate- or high-penetrance *BRCA1/2*, *RAD51C*, *RAD51D*, *BRIP1*, and mismatch repair genes. Cascade testing should be offered to relatives of carriers of gPVs. Gene sequencing can provide results with different biological meanings. A genetic alteration can be pathogenic or likely pathogenic, benign or likely benign, or finally of uncertain significance. The latter represents the main challenge when interpreting genetic alterations. In a large retrospective cohort of individuals who had genetics testing, 7.7% of unique gPVs of uncertain significance were reclassified. The majority of them (91.2%) were considered benign or likely benign [25]. Similarly, in a study on reinterpretation of BRCA1/2 gPVs of uncertain significance, 93.7% of the reclassified variants were benign or likely benign [26]. Despite this, gPVs of uncertain significance can be a great source of distress for patients and their families.

Although less prevalent, some non-epithelial ovarian cancers also have their risk enhanced by genetic factors. *DICER1* syndrome, characterized by germline truncating *DICER1* mutations includes predisposition to pleuropulmonary blastoma and Sertoli–Leydig cell tumors [27]. Germline mutations in *STK11* cause Peutz–Jeghers syndrome and are also associated with SCSTs [28]. 

## 3. Clinical Development of PARP Inhibitors

PARP inhibitors were originally developed for cancer treatment as radio- and chemosensitizing drugs. In 2014, the EMA approved a capsule formulation of olaparib as maintenance treatment in platinum-sensitive, *BRCA*-mutated (germline and/or somatic), high-grade serous EOC (stydy 19, NCT00753545) [29]. A few months later, the FDA approved olaparib capsules for the treatment of patients with deleterious or suspected deleterious germline *BRCA*-mutated advanced EOC, who have been treated with three or more prior lines of chemotherapy (stydy 42, NCT01078662) [30]. A tablet formulation of olaparib with improved bioavailability has been developed to facilitate olaparib administration to patients. It was approved by both agencies for the maintenance therapy of platinum-sensitive recurrent EOC regardless of *BRCA* mutational status (SOLO-2, NCT01874353) [29,31]. FDA approval of olaparib maintenance treatment in 2018 was supported by the SOLO-1 trial (NCT01844986), examining the efficacy of olaparib vs. placebo in patients with *BRCA*-mutated advanced EOC who responded to first-line platinum-based chemotherapy [32]. 

Rucaparib was approved by the FDA in 2016 and by the EMA in 2018 for patients with deleterious *BRCA* mutation (germline and/or somatic)-associated EOC who have been treated with two or more lines of chemotherapy. The efficacy was supported by a pooled analysis of two single-arm clinical trials: study 10 (NCT01482715) and ARIEL 2 (NCT01891344) [33,34,35]. ARIEL 2 enrolled platinum-sensitive EOC patients, assigned to one of three HR deficiency categories assessed on the most recent collected tumor sample: *BRCA1/2* mutated, *BRCA* wild-type (*BRCA*wt) with LOH high, and *BRCA*wt with LOH low, respectively [32,34]. The median progression-free survival (PFS) was significantly longer in the BRCA-mutated subgroup (12.8 months; HR = 0.27, *p* < 0.0001) and in the *BRCA*wt/LOH high (5.7 months; HR = 0.62, *p* = 0.011), as compared to the *BRCA*wt/LOH low subgroup (5.2 months). Similarly, the objective response rate was higher in the *BRCA*1/2 mutated and *BRCA*wt/LOH high subgroups, than the *BRCA*wt/LOH low subgroup (80%, 29%, and 10%, respectively). Genomic LOH was shown to be a better predictor of response to rucaparib in patients with *BRCA*wt tumors with a sensitivity of 78%, compared to mutations in other HR deficiency genes (sensitivity 11%, *p* < 0.0001) or methylation of *BRCA1* or *RAD51C* (sensitivity 48%, *p* < 0.021). However, by combining mutations in HR deficiency genes and methylation of *BRCA1* or *RAD51C*, no statistically different sensitivity was observed (sensitivity 59%, *p* = 0.13). 

In March 2017, the FDA approved niraparib as maintenance treatment of recurrent EOC in responders to platinum-based chemotherapy (NOVA trial, NCT01847274) [36]. Equally, in November 2017, the EMA approved niraparib for the maintenance treatment of patients with platinum-sensitive relapsed high-grade serous EOC who responded to platinum-based chemotherapy. In October 2019, the FDA approved niraparib for patients with advanced HR-deficient EOC treated with at least three prior chemotherapy regimens based on the results of the Quadra trial (NCT02354586) [37]. 

Apart from the already approved PARP inhibitors for the treatment of EOC in different settings, veliparib and talazoparib are in earlier clinical development [38,39]. Veliparib was evaluated mainly combined with chemotherapy or targeted agents, whilst at least in vitro talazoparib demonstrates more potent anti-tumor activity based on its enhanced PARP-DNA trapping ability.

Although PARP inhibitors oppose the catalytic activity of PARP in general, there are remarkable differences in their abilities to trap PARP based on the size and structure of each separate molecule [40]. This results in significant differences in doses among PARP inhibitors. It has been demonstrated that PARP trapping is associated with PARP inhibitor cytotoxic activity.

Recently, combination therapy of PARP and immune checkpoint inhibitors is being developed based on the immuno-regulatory effects of PARP inhibition. MEDIOLA (NCT02734004) is a phase II study of olaparib and durvalumab in patients with relapsed, platinum-sensitive, BRCA-mutated EOC, which reported an overall response rate of 72% [41]. The phase I/II TOPACIO study (NCT02657889) investigated the combination of niraparib and pembrolizumab in patients with platinum resistant/refractory EOC [42]. The overall response and the disease control rates were 18% and 65%, respectively. There was no difference in response by *BRCA* and HR deficiency status. 

## 4. AURKs in Ovarian Cancer

AURKs are serin-threonine kinases, which act as molecular switches, regulating multiple processes in cell division including spindle organization, chromosome alignment, the spindle assembly checkpoint, cytokinesis, and the abscission checkpoint [43]. The family of AURKs includes *aurora kinase A* (*AURKA*, STK15), *aurora kinase B* (*AURKB*, STK12), and *aurora kinase C* (*AURKC*, STK13) [43,44]. AURKs contain an N-terminal domain (39–139 aa), a kinase domain (250–300 aa), and a C-terminal domain (15–20 aa) [45]. *AURK* overexpression is common in EOC and has been correlated with prognostic value. 

There is enough evidence of cross-talk between AURKs and *p53*. Furthermore, AURKA interacts with proteins involved in apoptosis, specifically *p73*, a protein of the family of *p*53, implicated in the regulation of cell cycle and apoptosis. An in vitro study demonstrated that inhibition of *AURKA* in a *p53*-deficient cell line results in the expression of genes associated with *p73*-mediated apoptosis [46]. 

DNA damage induces activation of checkpoint kinase 1 (*CHEK1*), which then transduces the checkpoint signal and facilitates cell cycle arrest and DNA damage repair. It has been described as a synergistic anti-tumoral effect between AURKA and CHEK1 inhibitors in EOC [47]. Expression of *AURKA* and *CHEK1* has been associated with dismal prognosis in early-stage EOC. Based on that, molecular analyses of the pathways in which these genes participate may be used to select patients for whom AURKA inhibitors would be effective. 

Within the context of AURK inhibition in EOC, a large cohort of 240 patients with recurrent high-grade EOC who were referred to the phase I clinical trials program has been analyzed retrospectively [48]. Targeted agents included bevacizumab, vascular endothelial growth factor (VEGF) receptor inhibitors, and other compounds targeting the PI3K/AKT/mTOR, MAPK, Src, Wee1, and AURKA signaling pathways. Chemotherapy plus bevacizumab-based or AURKA kinase inhibitor-based regimens were potentially effective and yielded a median PFS of more than 6 months, which is indicative of potential benefit deriving from AURKA inhibitors. A preclinical study in EOC cell lines demonstrated that the selective small inhibitor alisertib inhibits epithelial–mesenchymal transition via the PI3K/AKT/mTOR and sirtuin-1 mediated pathways [49]. The selectivity in the inhibition of *AURKA* may be related to a more favorable toxicity profile and a better therapeutic index than pan-AURK inhibitors.

In vitro inhibition of *AURKA* with alisertib decreased the expression of PARP and *BRCA1/2* and stimulated the NHEJ repair pathway [50]. Following these findings, in vivo studies confirmed that AURKA inhibition elevated phosphorylated DNA-PKcs and decreased PARP levels. Alisertib treatment alone or combined with paclitaxel significantly reduced the growth and dissemination of orthotopic EOC xenografts. The potent synergy between alisertib and paclitaxel in vitro suggests that the combination of these agents may be more effective than either drug alone [51]. 

ENMD-2076 has selective activity against *AURKA*, as well as kinases involved in angiogenesis [52]. The rationale for a phase II trial of ENMD-2076 in OCCC was that apart from the strong expression of *VEGF* in this histological subtype, the overexpression of *AURKA* had also been associated with chemoresistance [53,54]. 

The pan-AURK inhibitor danusertib hydrochloride shows a dominant AURKB kinase inhibition-related cellular phenotype and mechanism of action in cells in vitro and in vivo. In a phase I trial, 56 patients received escalating doses of danusertib (24 h infusion every 14 days) without granulocyte colony-stimulating factor (G-CSF). Among them, one patient with refractory EOC had 27% tumor regression and 30% biochemical response, suggesting a potential activity in this setting [55]. 

Finally, the pan-AURK inhibitor tozasertib not only causes cytokinesis defects through AURK inhibition but is also a potent inhibitor of necroptosis, a cell death process regulated and executed by the RIPK1, RIPK3, and MLKL signaling axis. Tozasertib may enhance carboplatin activity by MTT proliferative assay in both platinum-sensitive and platinum-resistant EOC cell lines, regardless of p53 status [56]. A low dose of tozasertib promotes paclitaxel-induced apoptosis and is effective in paclitaxel-resistant cells [57]. Furthermore, the combination of tozasertib with valproic acid led to a synergistic effect on gynecologic cancer cells [58]. 

## 5. Metformin and Ovarian Cancer

Metformin was officially introduced to diabetes treatment in 1957 and still represents a well-recognized therapeutic choice for type 2 diabetes [59]. The preclinical anti-cancer efficacy of metformin has been demonstrated mainly in breast cancer [60]. In vitro studies have demonstrated that metformin may stimulate AMP-activated protein kinase activation in breast cancer cells, with inhibition of the mTOR pathway [61]. In this regard, several inhibitors of the PI3K/Akt pathway are under investigation in animal models and clinical trials in the field of breast cancer [62,63].

Similarly, metformin inhibits the AMPK-dependent growth of multiple EOC cell lines and inhibits several receptor tyrosine kinases, such as human epidermal growth factor receptor 4 (HER4), epidermal growth factor receptor (EGFR), and platelet-derived growth factor receptor (PDGF-R) [64]. Treatment with metformin in vitro and in vivo in murine experiments resulted in decreased angiogenesis in metastatic tissues and attenuated ovarian cancer cell adhesion [65,66]. It has also been demonstrated that the reduction in neovascularization following metformin treatment can be driven by blockage of the mTOR signaling pathway [67,68]. Furthermore, metformin targets ALDH+ EOC stem cell populations in vitro, leading to suppressed angiogenesis, proliferation, and tumor growth [69]. 

Programmed cell death is mediated by several protein factors that include the Bcl-2 protein family and the caspase group of cysteine proteases. The upregulation of the Bax (Bcl-2 family member) increases the activity of the caspases and enhances the apoptotic activity. The inhibition of caspase-3 is included in the mechanism by which insulin promotes apoptosis. Apart from Bax, insulin downregulates Bad, which prevents programmed cell death. Many preclinical EOC studies correlate Bcl-2-regulated apoptosis to metformin’s chemosensitizing effects [65,70,71]. The chemosensitizing effect of metformin seems to be correlated with p53 function. In the presence of *p53*, metformin suppresses hexokinase II (glycolytic enzyme) and pyruvate dehydrogenase kinase (anti-apoptotic serine/threonine kinase) [72]. As a result, EOC cells are sensitized to metformin. Furthermore, metformin may re-sensitize platinum- or paclitaxel-resistant EOC cells to chemosensitive cells, either by induction of autophagy or via its anti-inflammatory properties [67,73]. 

While metformin has wide anti-cancer effects in preclinical models, results of studies evaluated the association between metformin use and survival in ovarian cancer patients with type 2 diabetes are inconclusive [74,75,76,77,78]. So far, sufficient evidence verifying metformin use in ovarian cancer prevention is lacking. Register-based epidemiological studies of the incidence of ovarian cancer in patients taking metformin are depicted in Table 3.

## 6. Endometriosis and Ovarian Cancer Risk 

Both endometriosis and ovarian cancer have certain pathogenic similarities, and share similar risk factors [82]. The prevalence of ovarian cancer that appears in patients with endometriosis is greater than sporadic ovarian cancer in the general population. Women with endometriosis have an increased risk for certain subtypes of EOC, such as OCCC and EnOC [83,84]. 

There is strong evidence of a genetic link between endometriosis and ovarian cancer. Mutations in *ARID1A* have been found in several cancers, and SWI/SNF-associated gene mutations occur in approximately 20% of all malignancies, whereas the most frequent mutations in *ARID1A* are found in OCCC (46–57%) and EnOC (approx. 30%) [85,86,87,88]. Mutations in *ARID1A* result in the loss of BRG-associated factor 250a (BAF250a), a protein with an important role in cell proliferation and tumor suppression. It has been shown that loss of BAF250a presumably occurs at an early stage in carcinogenesis, as has been observed in a subset of benign endometriotic cysts of the ovary and deep-infiltrating endometriosis. Samartzis et al. described a complete loss of BAF250a in benign endometriotic lesions. Interestingly, the stromal BAF250a expression was lower in ovarian endometriosis, as compared to eutopic endometrium, peritoneal endometriosis, and deep-infiltrating endometriosis [89,90,91]. Identification of synthetic lethal targets that are conferred by these SWI/SNF-associated mutations on cancer cells requires further investigation and has important therapeutic potential. Targeting of sustained proliferative pathways, such as the PI3K/AKT/mTOR and the YES1/SRC tyrosine kinase pathways, or metabolic alterations, such as the glutathione biogenesis pathway, in *ARID1A*-deficient OCCC should be considered in future clinical trials. Such synthetic lethal agents in the *ARID1A* mutant setting are currently in clinical development. The inhibitory effects on residual SWI/SNF function, specifically via reduced ARID1B expression, may explain the enhanced sensitivity of *ARID1A* mutant cells to bromodomain and extraterminal domain (BET) inhibitors. As such, patients with *ARID1A* mutant OCCC may benefit from inhibitors of the BET family of proteins added to their treatment regimen. 

The discovery of frequent somatic phosphatase and tensin homolog (*PTEN*) mutations and loss of heterozygosity at the 10q23 *PTEN* locus in EnOC, along with an absence of such mutations in other histological subtypes, suggests a key role for *PTEN* in the etiology of this subtype. Using a mouse model, Dinulescu et al. induced EnOC and saw that expression of oncogenic *KRAS* or conditional *PTEN* deletion within the ovarian surface epithelium forms endometriotic precursor lesions. These alterations led to the development of invasive EnOC [92]. 

EZH2 is a histone methyltransferase that sets the H3K27me3 histone mark, a repressor of the transcriptional machinery. EZH2 also enhances angiogenesis, with a key role in ovarian carcinogenesis [93]. Higher levels of EZH2 correlated to a worse prognosis for EOC patients [94]. Due to in vivo-detected toxicity of first-generation EZH2 inhibitors, novel EZH2 inhibitors are currently investigated [95]. The NRG-GY-014 phase II clinical trial assessing the EZH2-inhibitor tazemetostat in recurrent EnOC, OCCC, and/or recurrent low-grade endometrioid endometrial carcinoma is currently recruiting [96]. 

There are several studies where differential expression of miRNAs has been studied in either endometriosis or ovarian cancer. Several differentially expressed miRNA in endometriosis compared to ovarian cancer have been found, mainly linked with epithelial–mesenchymal transition [97]. Two common miRNAs overexpressed in both diseases were miR-325 and miR-492. While the expression of miR-325 was upregulated in both diseases, this was more prominent in ovarian cancer, suggesting that miR-325 could have a role in the transition from endometriosis to ovarian cancer [97]. 

Among well-investigated epigenetic drugs in the field of ovarian cancer are histone deacetyltransferase (HDAC) inhibitors, which work by increasing the level of acetylated histones thus reactivating silenced tumor suppressor genes. Currently, only three HDAC inhibitors—vorinostat, romidepsin, and panobinostat—have been approved by the FDA [98]. HDAC inhibitors seem to be effective particularly in combination with other anti-cancer drugs and/or radiotherapy. A combination of DNA methyltransferases (DNMTs) and HDAC inhibitors has been shown to overcome platinum resistance in ovarian cancer. It has been demonstrated that the DNMT inhibitor decitabine may lead to demethylation of many genes, including *BRCA1* [99]. 

Hydralazine is a non-nucleoside DNA-demethylating drug with an anti-hypertensive effect. The mechanism of action of hydralazine is still a controversial issue, as some groups claimed that it binds to the catalytic site of DNMTs, while others reported that it reduces DNMT1 and DNMT3a expression via the extracellular signal-regulated kinase (ERK) pathway inhibition. This drug, combined with valproic acid, was assessed in refractory solid tumors, including ovarian cancer [100]. 

Another combination approach with good response refers to epigenetic inhibitors and immunotherapy. In a mouse model of EOC, DNMT and HDAC inhibitors improved the response to immune checkpoint therapy. Specifically, the DNMT inhibitor 5-azacytidine increased the number of CD45+ immune cells and the percentage of natural killer cells and active CD8+ cells in the microenvironment. As a result, tumor burden was reduced and survival was prolonged. A triple combination therapy consisting of a DNMT, HDAC, and an immune checkpoint inhibitor provided the greatest efficacy [101].

An overview of current clinical trials mainly regarding OCCC using an *ARID1A*-related treatment approach is available in Table 4. 

## 7. Management of Malignant Ovarian Germ Cell Tumors (MOGCTs)

Non-epithelial ovarian cancers are a group of uncommon, histologically, and clinically distinct tumors, with favorable prognosis as compared with the majority of their epithelial counterparts [102]. The two most frequently diagnosed non-epithelial ovarian cancers are MOGCTs and SCSTs, with several histological subtypes [103]. SCSTs arise from the sex cord and ovarian stroma and comprise granulosa cell tumors—the most common subtype, subdivided into juvenile and adult types—Sertoli–Leydig cell tumors, theca cell tumors and rare SCSTs with annular tubules. Ovarian small-cell cancers (hypercalcemic and non-hypercalcemic types) and sarcomas are extremely rare and aggressive cancers with dismal prognosis [27,104]. 

MOGCTs account for only 2–5% of all ovarian cancers. They typically occur in children and young women aged 10–30 years, with a peak incidence in the teenage years [105]. Their rarity in postmenopausal women can cause initial diagnostic uncertainty and lead to delayed or suboptimal treatment [106]. MOGCTs are divided into dysgerminomas and non-dysgerminomas including primarily yolk sac tumors and immature teratomas. The presence of bilateral ovarian involvement suggests dysgerminoma or mixed histology, with a predominant dysgerminoma element. Signs and symptoms of MOGCTs usually include abdominal pain with a palpable pelvic abdominal mass (85%), followed by abdominal distension (35%), fever and vaginal bleeding (10% each). Those patients may also exhibit symptoms of pregnancy or precocious puberty, related to β-human chorionic gonadotropin production by the tumor. Adverse prognostic factors include advanced-stage, residual tumor after salvage surgery, non-dysgerminoma histology, as well as elevated Ca125 and age > 40 years at initial diagnosis. Even advanced/metastatic disease is potentially curable, at least in 75% of cases [103]. 

Surgical staging remains the cornerstone in the management of MOGTs. Surgical procedures include exploratory laparotomy, peritoneal washing, omental biopsy, unilateral oophorectomy, and selective removal of enlarged lymph nodes. Hysterectomy and bilateral salpingo-oophorectomy and can be considered in patients who do not wish to preserve fertility [107]. This is not always feasible, given that MOGCTs typically affect women of childbearing age. Approximately 60–70% of MOGCTs are diagnosed at stage I. These tumors can be cured without postoperative chemotherapy. Fertility sparing surgery can be also proposed in advanced stages after careful discussion with young patients who desire pregnancy [108]. In the case of residual teratoma, second-look surgery is therapeutically indicated [109]. Current approaches to the treatment are summarized in Table 5.

The bleomycin/etoposide/cisplatin (BEP) regimen is the preferred adjuvant chemotherapy. There is consensus that three cycles of BEP prevent recurrence in cases with completely resected disease. Four to five cycles are recommended for patients with macroscopic residual disease; nevertheless, this should be continued for up to six cycles in those with ongoing radiological or biochemical response [103]. The ongoing chemotherapy phase 3 trials, summarized in Table 6, may change the clinical management of MOGCTs. The long-term side effects of platinum-based chemotherapy for MOGCTs are mostly irreversible and the severity is related to the chemotherapy cumulative dose. Identification of patients more likely to experience cisplatin-related toxicities could be based on several single-nucleotide polymorphisms [110]. 

Recurrences usually occur within two years of initial diagnosis and typically relapse peritoneal cavity and retroperitoneal lymph nodes. The salvage rate for chemotherapy in patients with MOGCTs is approximately 50%, and recommended regimens include vinblastine, ifosfamide, and cisplatin; etoposide, ifosfamide, and cisplatin; and paclitaxel, ifosfamide, and cisplatin [111,112]. Secondary cytoreductive surgery could be performed in selected patients with recurrent disease. 

Somatic mutations in MOGCTs are not a frequent phenomenon. The low mutation rate, the *p*53 wild-type signature, and other somatic copy number aberrations support the resemblance of MOGCTs to testicular germ cell tumors. An analysis of 87 MOGCTs identified recurrent mutations in *KIT* and *KRAS*, along with frequent focal amplifications of *PIK3CA* and *AKT1* in yolk sac tumors [113]. However, the clinical efficacy of any targeted treatment has not been reported in unselected patient populations. The lack of efficacy of imatinib in MOGCTs is probably related to the frequent mutations in the KIT enzymatic site, which leads to reduced sensitivity to imatinib blockade [114]. Further investigation of CDK4/6 inhibition for the treatment of teratoma is required based on the preliminary results indicating the safety and potential clinical benefit [115]. Similarly, immune checkpoint inhibitors in MOGCTs need to be unraveled [115,116].

## 8. Conclusions and Future Perspectives

Since inflammatory and epigenetic processes play a predominant role in the pathogenesis of endometriosis-associated ovarian carcinomas, immunotherapy as well as epigenetic treatment approaches open the way to more personalized and adaptive therapies. A combination of multiple biomarker changes rather than a single gene or marker is involved in the initiation and progression of either disease. Genetic risk has an important impact on ovarian cancer. The evolution of NGS allows a rapid evaluation of multiple cancer susceptibility genes at similar costs to single gene sequencing. Germline genetic testing should be offered to all newly diagnosed patients with EOC to detect gPVs in all genes associated with EOC susceptibility. Furthermore, tumor sequencing to identify potentially targetable somatic mutations is increasingly being used in high-grade serous EOC and influences decisions on patient treatment. HR deficiency remains a strong predictor of clinical benefit from PARP inhibitors. Beyond germline, PARP inhibitors may be effective in somatic *BRCA1/2* mutations as well. Among other tumors, high-grade serous EOCs have shown a high frequency of phenotypes with a gain of function of *AURK* and a loss of function of *p53*. An understanding of the functional diversity of AURKs could help to evaluate their relevance as potential therapeutic targets. Anti-mitotic, anti-angiogenic, and anti-inflammatory effects of metformin are well studied in vitro. Ongoing clinical trials will potentially clarify the role of metformin in ovarian cancer treatment. MOGCTs are rare entities, treated with surgery and possibly platinum-based regimens based on the stage of the disease. Preclinical work on MOGCTs is warranted to allow investigation of novel drug targets.

## Figures and Tables

**Table 1:** Impact of moderate- and high-penetrance genes for EOC.

Gene	Histologic Subtype	Frequency of Germline Pathogenic Variants	Lifetime Risk of EOC
*BRCA1*	HGSOC	3–15%	39–63%
*BRCA2*		3–6%	17–27%
*RAD51C*		0–2%	5.2–9%
*RAD51D*		0–1%	10–12%
*BRIP1*		0–2%	5.8%
MMR genes (*MLH1*, *MSH2*, *MSH6*, *PMS2*)	1. Endometrioid; 2. Clear-cell	0–1%	4–12%

EOC: epithelial ovarian cancer; HGSOC: high-grade serous ovarian cancer; MMR: mismatch repair.

**Table 2:** Clinical trials of EOC patients with homologous recombination (HR) deficiency.

Study	Population	Treatment Arms	Median PFS	HR	*p*
SOLO-1	Newly diagnosed stage III/IV high-grade EOC, *BRCA1/2* mutated, maintenance setting	Arm A: Olaparib Arm B: Placebo	PFS rate at 3 y, Arm A: 69% Arm B: 35%	0.28	<0.001
SOLO-2	Platinum-sensitive relapsed EOC, *BRCA1/2* mutated, maintenance setting	Arm A: Olaparib Arm B: Placebo	Arm A: 19.1 m Arm B: 5.5 m	0.30	<0.0001
SOLO-3	Recurrent EOC, g*BRCA*m	Arm A: Olaparib Arm B: CTH of physician’s choice	Arm A: 13.4 m Arm B: 9.2 m	0.62	0.013
NOVA	Recurrent EOC, previous response to platinum-based CTH, maintenance setting	Arm A: Niraparib Arm B: Placebo	g*BRCA*m cohort, Arm A: 21 m Arm B: 5.5 m	0.27	<0.001
			Non-g*BRCA*m cohort with HRD, Arm A: 12.9 m Arm B: 3.8 m	0.38	<0.001
			Overall non-g*BRCA*m cohort, Arm A: 9.3 m Arm B: 3.9 m	0.45	<0.001
ARIEL 3	Recurrent EOC, previous response to platinum-based CTH, maintenance setting	Arm A: Rucaparib Arm B: Placebo	Patients with *BRCA* mutations, Arm A: 16.6 m Arm B: 5.4 m	0.23	<0.0001
			Patients with HRD, Arm A: 13.6 m Arm B: 5.4 m	0.32	<0.0001
			ITT population, Arm A: 10.8 m Arm B: 5.4 m	0.36	<0.0001
PAOLA 1	Newly diagnosed stage III/IV high-grade EOC, *BRCA1/2* mutated, responders to first line platinum-taxane CTH + bevacizumab	Arm A: Bevacizumab + olaparib Arm B: Bevacizumab + placebo	Overall population, Arm A: 22.1 m Arm B: 16.6 m	0.59	<0.001
			Patients with HRD, including those with *BRCA* mutations, Arm A: 37.2 m Arm B: 17.7 m	0.33	
			Patients with HRD, without *BRCA* mutations, Arm A: 28.1 m Arm B: 16.6 m	0.43	

EOC: epithelial ovarian cancer; PFS: progression-free survival; y: year; m: month; CTH: chemotherapy; HRD: homologous recombination deficiency; HR: hazard ratio; ITT: intention to treat population.

**Table 3:** Incidence of ovarian cancer in metformin users among women with type 2 diabetes.

Publication Reference	Design	Cancer Patients (*n*)	Ovarian Cancer Patients (*n*)	Metformin Users (*n*)	Reference Group	Outcome
[79]	Case-control analysis	1611	85	41	Women with T2D and no prior metformin use	OR 0.38 (95% CI 0.18–0.81) when ≥10 prescriptions of metformin; OR 0.59 (95% CI 0.25–1.41) when <10 prescriptions of metformin
[80]	Observational study	479,475	3201	601	Women with T2D and no use of metformin	aHR 0.66 (95% CI 0.59–0.73); *p* < 0.01
[81]	Retrospective cohort study	NA	303	303	Women with T2D using other oral ADM	Full cohort aHR 1.02 (95% CI 0.72–1.45); Case-control aHR 0.91 (95% CI 0.61–1.34)

OR: odds ratio; CI: confidence interval; aHR: adjusted hazard ratio; ADM: anti-diabetic medication.

**Table 4:** Clinical trials in gynecological cancer using an *ARID1A*-related treatment approach (www.clinicaltrials.gov).

Study/Status	Phase	Patients *n*	Agent	Design	Primary Outcome
NCT04065269/Recruiting	II	40	AZD6738 (ATR inhibitor) + olaparib	Experiment 1A: AZD6738; relapsed ovarian (fallopian tube/primary peritoneal) and endometrial clear cell carcinomas with loss of *ARID1A* expression Experiment 1B: AZD6738 + olaparib; (depending on response rate in cohort 1A); relapsed ovarian (fallopian tube/primary peritoneal) and endometrial clear cell carcinomas with loss of *ARID1A* expression Experiment 2: AZD6738 + olaparib; relapsed ovarian (fallopian tube/primary peritoneal) and endometrial clear cell carcinomas without loss of *ARID1A* expression Experiment 3: AZD6738 + olaparib; relapsed rare gynecological cancers (endometrioid ovarian carcinoma, endometrioid endometrial carcinoma, cervical adenocarcinoma, cervical squamous, ovarian carcinosarcoma and endometrial carcinosarcoma) regardless of *ARID1A* status	ORR
NCT02059265/Active, not recruiting	II	35	Dasatinib	Dasatinib OD, days 1–28, until PD or unacceptable toxicity; endometrial clear cell adenocarcinoma; ovarian clear cell cystadenocarcinoma; recurrent fallopian tube carcinoma; recurrent ovarian carcinoma; recurrent primary peritoneal carcinoma; recurrent uterine corpus carcinoma;	ORR
NCT03297424/Recruiting	I/II	166	PLX2853 (BRD4 inhibitor)	phase 1b (dose escalation): up to 30 subjects with advanced malignancies phase 2a (dose expansion): 5 expansion cohorts in total; expansion cohorts 1–4: either 10 or 29 subjects per cohort: advanced SCLC, uveal melanoma, OCCC, and any other advanced malignancy with a known *ARID1A* mutation; expansion cohort 5: up to 20 subjects may be enrolled for NHL	1. AEs; 2. Pharmacokinetics of PLX2853 (AUC, Cmax, Tmax, t1/2); 3. Dose-limiting toxicity; 4. Response by RECIST 1.1 (solid tumors) or Lugano criteria (NHL)
NCT03348631/Suspended	II	86	Tazemetostat (EZH2 inhibitor)	Tazemetostat BID, days 1–28, until PD or unacceptable toxicity; FIGO grade 1/2 endometrial endometrioid; adenocarcinoma; recurrent endometrial endometrioid; adenocarcinoma; recurrent ovarian carcinoma; recurrent ovarian clear cell adenocarcinoma; recurrent ovarian endometrioid; adenocarcinoma; recurrent uterine corpus carcinoma	ORR
NCT01914510/Completed	II	40	ENMD-2076 (oral anti-angiogenic and anti-proliferative kinase inhibitor)	ENMD-2067 275 mg, OD, days 1–28; starting dose of 250 mg, OD, days 1–28 in subjects with a body surface area of less than 1.65 m^2^	1. 6 month PFS rate; 2. CR rate; 3. PR rate

ATR: ataxia telangiectasia and Rad3-related kinase; ORR: overall response rate; OD: once a day; PD: progressive disease; BRD4: bromodomain-containing protein 4; SCLC: small-cell lung cancer; OCCC: ovarian clear cell carcinoma; NHL: non-Hodgkin lymphoma; AEs: adverse events; AUC: area under the concentration–time curve; Cmax: maximum observed concentration; Tmax: time to peak concentration; t1/2: half-life; RECIST: Response Evaluation Criteria in Solid Tumors; EZH2: enhancer of zeste homolog 2; BID: twice a day; FIGO: International Federation of Gynecology and Obstetrics; PFS: progression-free survival; CR: complete response; PR: partial response.

**Table 5:** Management options for malignant ovarian germ cell tumors (MOGCTs).

	Dysgerminomas	Immature Teratomas	Other
Stage I	USO with preservation of the contralateral ovary and uterus for fertility; TAH-BSO is acceptable if childbearing has been completed		
Stage IA	No ACT	G1: No ACT	ACT
		G2: Consideration of ACT	
		G3: ACT	
Stage IB/C	ACT		
Stage II	USO with preservation of the contralateral ovary and uterus for fertility; TAH-BSO is acceptable if childbearing has been completed; ACT		
Stage III/IV	USO with preservation of the contralateral ovary and uterus for fertility; TAH-BSO is acceptable if childbearing has been completed; UDS-ACT; NACT-IDS when indicated		
Recurrent tumors	Palliative chemotherapy		

MOGCTs: malignant ovarian germ cell tumors; USO: unilateral salpingo-oophorectomy; TAH-BSO: total abdominal hysterectomy and bilateral salpingo-oophorectomy; ACT: adjuvant chemotherapy; G: grade; UDS: upfront debulking surgery; NACT: neoadjuvant chemotherapy; IDS: interval debulking surgery.

**Table 6:** Phase III trials of combination chemotherapy in MOGCTs still recruiting patients.

Study	Population	Patients *n*	Treatment Arms	Primary Endpoint
TIGER (NCT02375204)	Relapsed or refractory disease	420	Arm A: CDCT (TIP) Arm B: HDCT plus ASCT with TI-CE	OS
MOGCT-01 (NCT02429687)	Stage IIA-IVB, adjuvant treatment	129	Arm A: PT Arm B: BEP	PFS
ANZUP-1302 (NCT02582697)	Stage IV, intermediate or poor prognosis as defined by IGCCC classification	500	Arm A: accelerated BEP Arm B: standard BEP	PFS
NCT03067181	Low-risk stratum: *Age (years)*: <50 years; *Sites*: ovarian immature teratoma, GCT (all sites); *Stage*: Stage I Standard risk 1: *Age (years)*: <11; *Sites*: ovarian, testicular, or extragonadal site; *Stage*: FIGO stage II-IV; YST, EC, or choriocarcinoma Standard risk 2: *Age (years)*: >= 11 and <25; *Ovarian*: FIGO stage IC, II/III; YST, EC, or choriocarcinoma; *Testicular*: AJCC stage II/III, IGCCC good risk; YST, EC, or choriocarcinoma; *Extragonadal*: COG stage II; YST, EC, or choriocarcinoma	1680	Arm A: bleomycin/carboplatin/etoposide (up to 4 cycles) Arm B: BEP (up to 4 cycles) Arm C: bleomycin/carboplatin/etoposide (up to 3 cycles) Arm D: BEP (up to 3 cycles) Experiment (low risk): observation	1. OS; 2. PFS
NCT03418844	MOGCTs SCSTs Remission > 2 years following initial treatment	480	Self-questionnaires of living conditions and QoL; Interest group: patients treated with chemotherapy Control group: patients not treated with chemotherapy	1. Chronic fatigue; 2. Late sequelae of CTH (cardiac, pulmonary disorders); 3. QoL

MOGCTs: malignant ovarian germ cell tumors; CDCT: conventional-dose chemotherapy; TIP: paclitaxel/ifosfamide/cisplatin; HDCT: high-dose chemotherapy; ASCT: autologous stem cell transplant; TI-CE: paclitaxel plus ifosfamide followed by high-dose carboplatin and etoposide; OS: overall survival; PT: paclitaxel/cisplatin; BEP: bleomycin/etoposide/cisplatin; PFS: progression-free survival; IGCCC: international germ cell cancer consensus classification; GCT: germ cell tumors; FIGO: International Federation of Gynecology and Obstetrics; YST: yolk sac tumor; EC: embryonal carcinoma; AJCC: American Joint Committee on Cancer; COG: Children’s Oncology Group; SCSTs: sex cord-stromal cell tumors; QoL: quality of life; CTH: chemotherapy.

## References

[1] Siegel R.L., Miller K.D., Jemal A. (2020). Cancer statistics, 2020. CA Cancer. J. Clin..

[2] NCCN Clinical Practice Guidelines in Oncology (NCCN Guidelines^®^) Genetic/Familial High-Risk Assessment: Breast, Ovarian, and Pancreatic. https://www.nccn.org/professionals/physician_gls/pdf/genetics_bop.pdf.

[3] Boussios S., Moschetta M., Karihtala P., Samartzis E.P., Sheriff M., Pappas-Gogos G., Ozturk M.A., Uccello M., Karathanasi A., Tringos M. (2020). Development of new poly(ADP-ribose) polymerase (PARP) inhibitors in ovarian cancer: Quo Vadis?. Ann. Transl. Med..

[4] Boussios S., Karathanasi A., Cooke D., Neille C., Sadauskaite A., Moschetta M., Zakynthinakis-Kyriakou N., Pavlidis N. (2019). PARP Inhibitors in Ovarian Cancer: The Route to “Ithaca”. Diagnostics.

[5] Le D.T., Durham J.N., Smith K.N., Wang H., Bartlett B.R., Aulakh L.K., Lu S., Kemberling H., Wilt C., Luber B.S. (2017). Mismatch repair deficiency predicts response of solid tumors to PD-1 blockade. Science.

[6] Price K.S., Svenson A., King E., Ready K., Lazarin G.A. (2018). Inherited Cancer in the Age of Next-Generation Sequencing. Biol. Res. Nurs..

[7] Castéra L., Krieger S., Rousselin A., Legros A., Baumann J.J., Bruet O., Brault B., Fouillet R., Goardon N., Letac O. (2014). Next-generation sequencing for the diagnosis of hereditary breast and ovarian cancer using genomic capture targeting multiple candidate genes. Eur. J. Hum. Genet..

[8] Ronson G.E., Piberger A.L., Higgs M.R., Olsen A.L., Stewart G.S., McHugh P.J., Petermann E., Lakin N.D. (2018). PARP1 and PARP2 stabilise replication forks at base excision repair intermediates through Fbh1-dependent Rad51 regulation. Nat. Commun..

[9] Pommier Y., O’Connor M.J., de Bono J. (2016). Laying a trap to kill cancer cells: PARP inhibitors and their mechanisms of action. Sci. Transl. Med..

[10] Price B.D., D’Andrea A.D. (2013). Chromatin remodeling at DNA double-strand breaks. Cell.

[11] Capoluongo E., Ellison G., López-Guerrero J.A., Penault-Llorca F., Ligtenberg M.J.L., Banerjee S., Singer C., Friedman E., Markiefka B., Schirmacher P. (2017). Guidance Statement On BRCA1/2 Tumor Testing in Ovarian Cancer Patients. Semin. Oncol..

[12] Watkins J.A., Irshad S., Grigoriadis A., Tutt A.N. (2014). Genomic scars as biomarkers of homologous recombination deficiency and drug response in breast and ovarian cancers. Breast Cancer Res..

[13] Coleman R.L., Oza A.M., Lorusso D., Aghajanian C., Oaknin A., Dean A., Colombo N., Weberpals J.I., Clamp A., Scambia G. (2017). Rucaparib maintenance treatment for recurrent ovarian carcinoma after response to platinum therapy (ARIEL3): A randomised, double-blind, placebo-controlled, phase 3 trial. Lancet.

[14] Phelan C.M., Kuchenbaecker K.B., Tyrer J.P., Kar S.P., Lawrenson K., Winham S.J., Dennis J., Pirie A., Riggan M.J., Chornokur G. (2017). Identification of 12 New Susceptibility Loci for Different Histotypes of Epithelial Ovarian Cancer. Nat. Genet..

[15] Loveday C., Turnbull C., Ruark E., Xicola R.M., Ramsay E., Hughes D., Warren-Perry M., Snape K., Eccles D., Breast Cancer Susceptibility Collaboration (UK) (2012). Germline RAD51C mutations confer susceptibility to ovarian cancer. Nat. Genet..

[16] Daly M.B., Pilarski R., Axilbund J.E., Berry M., Buys S.S., Crawford B., Farmer M., Friedman S., Garber J.E., Khan S. (2016). Genetic/Familial High-Risk Assessment: Breast and Ovarian, Version 2.2015. J. Natl. Compr. Canc. Netw..

[17] Ramus S.J., Song H., Dicks E., Tyrer J.P., Rosenthal A.N., Intermaggio M.P., Fraser L., Gentry-Maharaj A., Hayward J., Philpott S. (2015). Germline Mutations in the BRIP1, BARD1, PALB2, and NBN Genes in Women With Ovarian Cancer. J. Natl. Cancer Inst..

[18] Yang X., Leslie G., Doroszuk A., Schneider S., Allen J., Decker B., Dunning A.M., Redman J., Scarth J., Plaskocinska I. (2020). Cancer Risks Associated With Germline PALB2 Pathogenic Variants: An International Study of 524 Families. J. Clin. Oncol..

[19] Seal S., Thompson D., Renwick A., Elliott A., Kelly P., Barfoot R., Chagtai T., Jayatilake H., Ahmed M., Spanova K. (2006). Truncating mutations in the Fanconi anemia J gene BRIP1 are low-penetrance breast cancer susceptibility alleles. Nat. Genet..

[20] Hitchins M.P., Ward R.L. (2009). Constitutional (germline) MLH1 epimutation as an aetiological mechanism for hereditary non-polyposis colorectal cancer. J. Med. Genet..

[21] Kato M., Takano M., Miyamoto M., Sasaki N., Goto T., Tsuda H., Furuya K. (2015). DNA mismatch repair-related protein loss as a prognostic factor in endometrial cancers. J. Gynecol. Oncol..

[22] Matthews K.S., Estes J.M., Conner M.G., Manne U., Whitworth J.M., Huh W.K., Alvarez R.D., Straughn J.M., Barnes M.N., Rocconi R.P. (2008). Lynch syndrome in women less than 50 years of age with endometrial cancer. Obstet. Gynecol..

[23] Ruiz I., Martín-Arruti M., Lopez-Lopez E., Garcia-Orad A. (2014). Lack of association between deficient mismatch repair expression and outcome in endometrial carcinomas of the endometrioid type. Gynecol. Oncol..

[24] Nelson G.S., Pink A., Lee S., Han G., Morris D., Ogilvie T., Duggan M.A., Köbel M. (2013). MMR deficiency is common in high-grade endometrioid carcinomas and is associated with an unfavorable outcome. Gynecol. Oncol..

[25] Mersch J., Brown N., Pirzadeh-Miller S., Mundt E., Cox H.C., Brown K., Aston M., Esterling L., Manley S., Ross T. (2018). Prevalence of Variant Reclassification Following Hereditary Cancer Genetic Testing. JAMA.

[26] So M.K., Jeong T.D., Lim W., Moon B.I., Paik N.S., Kim S.C., Huh J. (2019). Reinterpretation of BRCA1 and BRCA2 Variants of Uncertain Significance in Patients With Hereditary Breast/Ovarian Cancer Using the ACMG/AMP 2015 Guidelines. Breast Cancer.

[27] Boussios S., Moschetta M., Zarkavelis G., Papadaki A., Kefas A., Tatsi K. (2017). Ovarian sex-cord stromal tumours and small cell tumours: Pathological, genetic and management aspects. Crit. Rev. Oncol. Hematol..

[28] Young R.H., Dickersin G.R., Scully R.E. (1983). A distinctive ovarian sex cord-stromal tumor causing sexual precocity in the Peutz-Jeghers syndrome. Am. J. Surg. Pathol..

[29] Ledermann J., Harter P., Gourley C., Friedlander M., Vergote I., Rustin G., Scott C.L., Meier W., Shapira-Frommer R., Safra T. (2014). Olaparib maintenance therapy in patients with platinum-sensitive relapsed serous ovarian cancer: A preplanned retrospective analysis of outcomes by BRCA status in a randomised phase 2 trial. Lancet Oncol..

[30] Kaufman B., Shapira-Frommer R., Schmutzler R.K., Audeh M.W., Friedlander M., Balmaña J., Mitchell G., Fried G., Stemmer S.M., Hubert A. (2015). Olaparib monotherapy in patients with advanced cancer and a germline BRCA1/2 mutation. J. Clin. Oncol..

[31] Pujade-Lauraine E., Ledermann J.A., Selle F., Gebski V., Penson R.T., Oza A.M., Korach J., Huzarski T., Poveda A., Pignata S. (2017). Olaparib tablets as maintenance therapy in patients with platinum-sensitive, relapsed ovarian cancer and a BRCA1/2 mutation (SOLO2/ENGOT-Ov21): A double-blind, randomised, placebo-controlled, phase 3 trial. Lancet Oncol..

[32] Moore K., Colombo N., Scambia G., Kim B.G., Oaknin A., Friedlander M., Lisyanskaya A., Floquet A., Leary A., Sonke G.S. (2018). Maintenance Olaparib in Patients with Newly Diagnosed Advanced Ovarian Cancer. N. Engl. J. Med..

[33] Kristeleit R., Shapiro G.I., Burris H.A., Oza A.M., LoRusso P., Patel M.R., Domchek S.M., Balmaña J., Drew Y., Chen L.M. (2017). A Phase I-II Study of the Oral PARP Inhibitor Rucaparib in Patients with Germline BRCA1/2-Mutated Ovarian Carcinoma or Other Solid Tumors. Clin. Cancer Res..

[34] Swisher E.M., Lin K.K., Oza A.M., Scott C.L., Giordano H., Sun J., Konecny G.E., Coleman R.L., Tinker A.V., O’Malley D.M. (2017). Rucaparib in relapsed, platinum-sensitive high-grade ovarian carcinoma (ARIEL2 Part 1): An international, multicentre, open-label, phase 2 trial. Lancet Oncol..

[35] Oza A.M., Tinker A.V., Oaknin A., Shapira-Frommer R., McNeish I.A., Swisher E.M., Ray-Coquard I., Bell-McGuinn K., Coleman R.L., O’Malley D.M. (2017). Antitumor activity and safety of the PARP inhibitor rucaparib in patients with high-grade ovarian carcinoma and a germline or somatic BRCA1 or BRCA2 mutation: Integrated analysis of data from Study 10 and ARIEL2. Gynecol. Oncol..

[36] Del Campo J.M., Matulonis U.A., Malander S., Provencher D., Mahner S., Follana P., Waters J., Berek J.S., Woie K., Oza A.M. (2019). Niraparib Maintenance Therapy in Patients with Recurrent Ovarian Cancer after a Partial Response to the Last Platinum-Based Chemotherapy in the ENGOT-OV16/NOVA Trial. J. Clin. Oncol..

[37] Moore K.N., Secord A.A., Geller M.A., Miller D.S., Cloven N., Fleming G.F., Wahner Hendrickson A.E., Azodi M., DiSilvestro P., Oza A.M. (2019). Niraparib monotherapy for late-line treatment of ovarian cancer (QUADRA): A multicentre, open-label, single-arm, phase 2 trial. Lancet Oncol..

[38] Boussios S., Karihtala P., Moschetta M., Abson C., Karathanasi A., Zakynthinakis-Kyriakou N., Ryan J.E., Sheriff M., Rassy E., Pavlidis N. (2020). Veliparib in ovarian cancer: A new synthetically lethal therapeutic approach. Investig. New Drugs.

[39] Boussios S., Abson C., Moschetta M., Rassy E., Karathanasi A., Bhat T., Ghumman F., Sheriff M., Pavlidis N. (2020). Poly (ADP-Ribose) Polymerase Inhibitors: Talazoparib in Ovarian Cancer and Beyond. Drugs R D.

[40] Boussios S., Karihtala P., Moschetta M., Karathanasi A., Sadauskaite A., Rassy E., Pavlidis N. (2019). Combined Strategies with Poly (ADP-Ribose) Polymerase (PARP) Inhibitors for the Treatment of Ovarian Cancer: A Literature Review. Diagnostics.

[41] Drew Y., de Jonge M., Hong S.H., Park Y.H., Wolfer A., Brown J., Ferguson M., Gore M.E., Alvarez R.H., Grest C. (2018). An open-label, phase II basket study of olaparib and durvalumab (MEDIOLA): Results in germline BRCA-mutated (gBRCAm) platinum-sensitive relapsed (PSR) ovarian cancer (OC). Gynecol. Oncol..

[42] Konstantinopoulos P.A., Waggoner S.E., Vidal G.A., Mita M.M., Fleming G.F., Holloway R.W., Van Le L., Sachdev J.C., Chapman-Davis E., Colon-Otero G. (2018). TOPACIO/Keynote-162 (NCT02657889): A phase 1/2 study of niraparib + pembrolizumab in patients (pts) with advanced triple-negative breast cancer or recurrent ovarian cancer (ROC)—Results from ROC cohort. J. Clin. Oncol..

[43] Carmena M., Earnshaw W.C. (2003). The cellular geography of aurora kinases. Nat. Rev. Mol. Cell Biol..

[44] Pérez Fidalgo J.A., Roda D., Roselló S., Rodríguez-Braun E., Cervantes A. (2009). Aurora kinase inhibitors: A new class of drugs targeting the regulatory mitotic system. Clin. Transl. Oncol..

[45] Bolanos-Garcia V.M. (2005). Aurora kinases. Int. J. Biochem. Cell Biol..

[46] Katayama H., Wang J., Treekitkarnmongkol W., Kawai H., Sasai K., Zhang H., Wang H., Adams H.P., Jiang S., Chakraborty S.N. (2012). Aurora kinase-A inactivates DNA damage-induced apoptosis and spindle assembly checkpoint response functions of p73. Cancer Cell.

[47] Alcaraz-Sanabria A., Nieto-Jiménez C., Corrales-Sánchez V., Serrano-Oviedo L., Andrés-Pretel F., Montero J.C., Burgos M., Llopis J., Galán-Moya E.M., Pandiella A. (2017). Synthetic Lethality Interaction Between Aurora Kinases and CHEK1 Inhibitors in Ovarian Cancer. Mol. Cancer Ther..

[48] Hou M.M., Wang Z., Janku F., Piha-Paul S., Naing A., Hong D., Westin S., Coleman R.L., Sood A.K., Tsimberidou A.M. (2016). Continuous anti-angiogenic therapy after tumor progression in patients with recurrent high-grade epithelial ovarian cancer: Phase I trial experience. Oncotarget.

[49] Ding Y.H., Zhou Z.W., Ha C.F., Zhang X.Y., Pan S.T., He Z.X., Edelman J.L., Wang D., Yang Y.X., Zhang X. (2015). Alisertib, an Aurora kinase A inhibitor, induces apoptosis and autophagy but inhibits epithelial to mesenchymal transition in human epithelial ovarian cancer cells. Drug Des. Devel. Ther..

[50] Do T.V., Hirst J., Hyter S., Roby K.F., Godwin A.K. (2017). Aurora A kinase regulates non-homologous end-joining and poly (ADP-ribose) polymerase function in ovarian carcinoma cells. Oncotarget.

[51] Do T.V., Xiao F., Bickel L.E., Klein-Szanto A.J., Pathak H.B., Hua X., Howe C., O’Brien S.W., Maglaty M., Ecsedy J.A. (2014). Aurora kinase A mediates epithelial ovarian cancer cell migration and adhesion. Oncogene.

[52] Fletcher G.C., Brokx R.D., Denny T.A., Hembrough T.A., Plum S.M., Fogler W.E., Sidor C.F., Bray M.R. (2011). ENMD-2076 is an orally active kinase inhibitor with antiangiogenic and antiproliferative mechanisms of action. Mol. Cancer Ther..

[53] Lheureux S., Tinker A., Clarke B., Ghatage P., Welch S., Weberpals J.I., Dhani N.C., Butler M.O., Tonkin K., Tan Q. (2018). A Clinical and Molecular Phase II Trial of Oral ENMD-2076 in Ovarian Clear Cell Carcinoma (OCCC): A Study of the Princess Margaret Phase II Consortium. Clin. Cancer Res..

[54] Li M., Li H., Liu F., Bi R., Tu X., Chen L., Ye S., Cheng X. (2017). Characterization of ovarian clear cell carcinoma using target drug-based molecular biomarkers: Implications for personalized cancer therapy. J. Ovarian. Res..

[55] Cohen R.B., Jones S.F., Aggarwal C., von Mehren M., Cheng J., Spigel D.R., Greco F.A., Mariani M., Rocchetti M., Ceruti R. (2009). A phase I dose-escalation study of danusertib (PHA-739358) administered as a 24-h infusion with and without granulocyte colony-stimulating factor in a 14-day cycle in patients with advanced solid tumors. Clin. Cancer Res..

[56] Fu S., Li Y., Huang J., Liu T., Hong Z., Chen A., Bast R.C., Kavanagh J.J., Gershenson D.M., Sood A.K. (2012). Aurora kinase inhibitor VE 465 synergistically enhances cytotoxicity of carboplatin in ovarian cancer cells through induction of apoptosis and downregulation of histone 3. Cancer Biol. Ther..

[57] Scharer C.D., Laycock N., Osunkoya A.O., Logani S., McDonald J.F., Benigno B.B., Moreno C.S. (2008). Aurora kinase inhibitors synergize with paclitaxel to induce apoptosis in ovarian cancer cells. J. Transl. Med..

[58] Li Y., Liu T., Ivan C., Huang J., Shen D.Y., Kavanagh J.J., Bast R.C., Fu S., Hu W., Sood A.K. (2013). Enhanced Cytotoxic Effects of Combined Valproic Acid and the Aurora Kinase Inhibitor VE465 on Gynecologic Cancer Cells. Front. Oncol..

[59] Flory J., Lipska K. (2019). Metformin in 2019. JAMA.

[60] Aljofan M., Riethmacher D. (2019). Anticancer activity of metformin: A systematic review of the literature. Future Sci. OA.

[61] Dowling R.J., Zakikhani M., Fantus I.G., Pollak M., Sonenberg N. (2007). Metformin inhibits mammalian target of rapamycin-dependent translation initiation in breast cancer cells. Cancer Res..

[62] Gallagher E.J., Fierz Y., Vijayakumar A., Haddad N., Yakar S., LeRoith D. (2012). Inhibiting PI3K reduces mammary tumor growth and induces hyperglycemia in a mouse model of insulin resistance and hyperinsulinemia. Oncogene.

[63] Hernandez-Aya L.F., Gonzalez-Angulo A.M. (2011). Targeting the phosphatidylinositol 3-kinase signaling pathway in breast cancer. Oncologist.

[64] Lengyel E., Litchfield L.M., Mitra A.K., Nieman K.M., Mukherjee A., Zhang Y., Johnson A., Bradaric M., Lee W., Romero I.L. (2015). Metformin inhibits ovarian cancer growth and increases sensitivity to paclitaxel in mouse models. Am. J. Obstet. Gynecol..

[65] Dang J.H., Jin Z.J., Liu X.J., Hu D., Wang J., Luo Y., Li L.L. (2017). Metformin in combination with cisplatin inhibits cell viability and induces apoptosis of human ovarian cancer cells by inactivating ERK 1/2. Oncol. Lett..

[66] Wu B., Li S., Sheng L., Zhu J., Gu L., Shen H., La D., Hambly B.D., Bao S., Di W. (2012). Metformin inhibits the development and metastasis of ovarian cancer. Oncol. Rep..

[67] Erices R., Cubillos S., Aravena R., Santoro F., Marquez M., Orellana R., Ramírez C., González P., Fuenzalida P., Bravo M.L. (2017). Diabetic concentrations of metformin inhibit platelet-mediated ovarian cancer cell progression. Oncotarget.

[68] Liao H., Zhou Q., Gu Y., Duan T., Feng Y. (2012). Luteinizing hormone facilitates angiogenesis in ovarian epithelial tumor cells and metformin inhibits the effect through the mTOR signaling pathway. Oncol. Rep..

[69] Shank J.J., Yang K., Ghannam J., Cabrera L., Johnston C.J., Reynolds R.K., Buckanovich R.J. (2012). Metformin targets ovarian cancer stem cells in vitro and in vivo. Gynecol. Oncol..

[70] Yasmeen A., Beauchamp M.C., Piura E., Segal E., Pollak M., Gotlieb W.H. (2011). Induction of apoptosis by metformin in epithelial ovarian cancer: Involvement of the Bcl-2 family proteins. Gynecol. Oncol..

[71] Patel S., Singh N., Kumar L. (2015). Evaluation of Effects of Metformin in Primary Ovarian Cancer Cells. Asian Pac. J. Cancer Prev..

[72] Han C.Y., Patten D.A., Lee S.G., Parks R.J., Chan D.W., Harper M.E., Tsang B.K. (2019). p53 Promotes chemoresponsiveness by regulating hexokinase II gene transcription and metabolic reprogramming in epithelial ovarian cancer. Mol. Carcinog..

[73] Yang C., Zhao N., Li D., Zou G., Chen Y. (2019). Metformin improves the sensitivity of ovarian cancer cells to chemotherapeutic agents. Oncol. Lett..

[74] Kumar S., Meuter A., Thapa P., Langstraat C., Giri S., Chien J., Rattan R., Cliby W., Shridhar V. (2013). Metformin intake is associated with better survival in ovarian cancer: A case-control study. Cancer.

[75] Wang S.B., Lei K.J., Liu J.P., Jia Y.M. (2017). Continuous use of metformin can improve survival in type 2 diabetic patients with ovarian cancer: A retrospective study. Medicine.

[76] Romero I.L., McCormick A., McEwen K.A., Park S., Karrison T., Yamada S.D., Pannain S., Lengyel E. (2012). Relationship of type II diabetes and metformin use to ovarian cancer progression, survival, and chemosensitivity. Obstet. Gynecol..

[77] Garcia C., Yao A., Camacho F., Balkrishnan R., Cantrell L.A. (2017). A SEER-Medicare analysis of the impact of metformin on overall survival in ovarian cancer. Gynecol. Oncol..

[78] Urpilainen E., Marttila M., Hautakoski A., Arffman M., Sund R., Ilanne-Parikka P., Arima R., Kangaskokko J., Puistola U., Hinkula M. (2018). Prognosis of ovarian cancer in women with type 2 diabetes using metformin and other forms of antidiabetic medication or statins: A retrospective cohort study. BMC Cancer.

[79] Bodmer M., Becker C., Meier C., Jick S.S., Meier C.R. (2011). Use of metformin and the risk of ovarian cancer: A case-control analysis. Gynecol. Oncol..

[80] Tseng C.H. (2015). Metformin reduces ovarian cancer risk in Taiwanese women with type 2 diabetes mellitus. Diabetes Metab. Res. Rev..

[81] Urpilainen E., Marttila M., Hautakoski A., Arffman M., Sund R., Ilanne-Parikka P., Arima R., Kangaskokko J., Puistola U., Läärä E. (2018). The role of metformin and statins in the incidence of epithelial ovarian cancer in type 2 diabetes: A cohort and nested case-control study. BJOG.

[82] Sova H., Kangas J., Puistola U., Santala M., Liakka A., Karihtala P. (2012). Down-regulation of 8-hydroxydeoxyguanosine and peroxiredoxin II in the pathogenesis of endometriosis-associated ovarian cancer. Anticancer Res..

[83] Robinson K.A., Menias C.O., Chen L., Schiappacasse G., Shaaban A.M., Caserta M.P., Elsayes K.M., VanBuren W.M., Bolan C.W. (2020). Understanding malignant transformation of endometriosis: Imaging features with pathologic correlation. Abdom. Radiol..

[84] Siufi Neto J., Kho R.M., Siufi D.F., Baracat E.C., Anderson K.S., Abrão M.S. (2014). Cellular, histologic, and molecular changes associated with endometriosis and ovarian cancer. J. Minim. Invasive Gynecol..

[85] Xu G., Chhangawala S., Cocco E., Razavi P., Cai Y., Otto J.E., Ferrando L., Selenica P., Ladewig E., Chan C. (2020). ARID1A determines luminal identity and therapeutic response in estrogen-receptor-positive breast cancer. Nat. Genet..

[86] Nagarajan S., Rao S.V., Sutton J., Cheeseman D., Dunn S., Papachristou E.K., Prada J.G., Couturier D.L., Kumar S., Kishore K. (2020). ARID1A influences HDAC1/BRD4 activity, intrinsic proliferative capacity and breast cancer treatment response. Nat. Genet..

[87] Wiegand K.C., Shah S.P., Al-Agha O.M., Zhao Y., Tse K., Zeng T., Senz J., McConechy M.K., Anglesio M.S., Kalloger S.E. (2010). ARID1A mutations in endometriosis-associated ovarian carcinomas. N. Engl. J. Med..

[88] Jones S., Wang T.L., Shih I.M., Mao T.L., Nakayama K., Roden R., Glas R., Slamon D., Diaz L.A., Vogelstein B. (2010). Frequent mutations of chromatin remodeling gene ARID1A in ovarian clear cell carcinoma. Science.

[89] Samartzis E.P., Samartzis N., Noske A., Fedier A., Caduff R., Dedes K.J., Fink D., Imesch P. (2012). Loss of ARID1A/BAF250a-expression in endometriosis: A biomarker for risk of carcinogenic transformation?. Mod. Pathol..

[90] Borelli G.M., Abrão M.S., Taube E.T., Darb-Esfahani S., Köhler C., Chiantera V., Mechsner S. (2016). (Partial) Loss of BAF250a (ARID1A) in rectovaginal deep-infiltrating endometriosis, endometriomas and involved pelvic sentinel lymph nodes. Mol. Hum. Reprod..

[91] Anglesio M.S., Papadopoulos N., Ayhan A., Nazeran T.M., Noë M., Horlings H.M., Lum A., Jones S., Senz J., Seckin T. (2017). Cancer-Associated Mutations in Endometriosis without Cancer. N. Engl. J. Med..

[92] Dinulescu D.M., Ince T.A., Quade B.J., Shafer S.A., Crowley D., Jacks T. (2005). Role of K-ras and Pten in the development of mouse models of endometriosis and endometrioid ovarian cancer. Nat. Med..

[93] Bitler B.G., Aird K.M., Zhang R. (2015). Epigenetic synthetic lethality in ovarian clear cell carcinoma: EZH2 and ARID1A mutations. Mol. Cell Oncol..

[94] Li H., Zhang R. (2013). Role of EZH2 in Epithelial Ovarian Cancer: From Biological Insights to Therapeutic Target. Front. Oncol..

[95] Kim K.H., Roberts C.W. (2016). Targeting EZH2 in cancer. Nat. Med..

[96] Lee J.M., Minasian L., Kohn E.C. (2019). New strategies in ovarian cancer treatment. Cancer.

[97] Braicu O.L., Budisan L., Buiga R., Jurj A., Achimas-Cadariu P., Pop L.A., Braicu C., Irimie A., Berindan-Neagoe I. (2017). miRNA expression profiling in formalin-fixed paraffin-embedded endometriosis and ovarian cancer samples. Onco. Targets Ther..

[98] Yang Q., Yang Y., Zhou N., Tang K., Lau W.B., Lau B., Wang W., Xu L., Yang Z., Huang S. (2018). Epigenetics in ovarian cancer: Premise, properties, and perspectives. Mol. Cancer.

[99] Ding Y.B., Long C.L., Liu X.Q., Chen X.M., Guo L.R., Xia Y.Y., He J.L., Wang Y.X. (2012). 5-aza-2’-deoxycytidine leads to reduced embryo implantation and reduced expression of DNA methyltransferases and essential endometrial genes. PLoS ONE.

[100] Bauman J., Shaheen M., Verschraegen C.F., Belinsky S.A., Houman Fekrazad M., Lee F.C., Rabinowitz I., Ravindranathan M., Jones D.V. (2014). A Phase I Protocol of Hydralazine and Valproic Acid in Advanced, Previously Treated Solid Cancers. Transl. Oncol..

[101] Oki S., Sone K., Oda K., Hamamoto R., Ikemura M., Maeda D., Takeuchi M., Tanikawa M., Mori-Uchino M., Nagasaka K. (2017). Oncogenic histone methyltransferase EZH2: A novel prognostic marker with therapeutic potential in endometrial cancer. Oncotarget.

[102] Matz M., Coleman M.P., Carreira H., Salmerón D., Chirlaque M.D., Allemani C., CONCORD Working Group (2017). Worldwide comparison of ovarian cancer survival: Histological group and stage at diagnosis (CONCORD-2). Gynecol. Oncol..

[103] Boussios S., Zarkavelis G., Seraj E., Zerdes I., Tatsi K., Pentheroudakis G. (2016). Non-epithelial Ovarian Cancer: Elucidating Uncommon Gynaecological Malignancies. Anticancer Res..

[104] Boussios S., Karathanasi A., Zakynthinakis-Kyriakou N., Tsiouris A.K., Chatziantoniou A.A., Kanellos F.S., Tatsi K. (2019). Ovarian carcinosarcoma: Current developments and future perspectives. Crit. Rev. Oncol. Hematol..

[105] Smith H.O., Berwick M., Verschraegen C.F., Wiggins C., Lansing L., Muller C.Y., Qualls C.R. (2006). Incidence and survival rates for female malignant germ cell tumors. Obstet. Gynecol..

[106] Boussios S., Attygalle A., Hazell S., Moschetta M., McLachlan J., Okines A., Banerjee S. (2015). Malignant Ovarian Germ Cell Tumors in Postmenopausal Patients: The Royal Marsden Experience and Literature Review. Anticancer Res..

[107] Liu Q., Ding X., Yang J., Cao D., Shen K., Lang J., Zhang G., Xin X., Xie X., Wu Y. (2013). The significance of comprehensive staging surgery in malignant ovarian germ cell tumors. Gynecol. Oncol..

[108] Di Tucci C., Casorelli A., Morrocchi E., Palaia I., Muzii L., Panici P.B. (2017). Fertility management for malignant ovarian germ cell tumors patients. Crit. Rev. Oncol. Hematol..

[109] Zagamé L., Pautier P., Duvillard P., Castaigne D., Patte C., Lhommé C. (2006). Growing teratoma syndrome after ovarian germ cell tumors. Obstet. Gynecol..

[110] Uccello M., Boussios S., Samartzis E.P., Moschetta M. (2020). Systemic anti-cancer treatment in malignant ovarian germ cell tumours (MOGCTs): Current management and promising approaches. Ann. Transl. Med..

[111] Gershenson D.M. (2007). Management of ovarian germ cell tumors. J. Clin. Oncol..

[112] Kondagunta G.V., Bacik J., Donadio A., Bajorin D., Marion S., Sheinfeld J., Bosl G.J., Motzer R.J. (2005). Combination of paclitaxel, ifosfamide, and cisplatin is an effective second-line therapy for patients with relapsed testicular germ cell tumors. J. Clin. Oncol..

[113] Van Nieuwenhuysen E., Busschaert P., Neven P., Han S.N., Moerman P., Liontos M., Papaspirou M., Kupryjanczyk J., Hogdall C., Hogdall E. (2018). The genetic landscape of 87 ovarian germ cell tumors. Gynecol. Oncol..

[114] Einhorn L.H., Brames M.J., Heinrich M.C., Corless C.L., Madani A. (2006). Phase II study of imatinib mesylate in chemotherapy refractory germ cell tumors expressing KIT. Am. J. Clin. Oncol..

[115] Narayan V., Hwang W.T., Lal P., Rosen M.A., Gallagher M., O’Dwyer P.J., Vaughn D.J. (2016). Cyclin-Dependent Kinase 4/6 Inhibition for the Treatment of Unresectable Mature Teratoma: Long-Term Follow-Up of a Phase II Study. Clin. Genitourin. Cancer.

[116] Hong L.K., Chen Y., Smith C.C., Montgomery S.A., Vincent B.G., Dotti G., Savoldo B. (2018). CD30-Redirected Chimeric Antigen Receptor T Cells Target CD30^+^ and CD30^−^ Embryonal Carcinoma via Antigen-Dependent and Fas/FasL Interactions. Cancer Immunol. Res..

